# Polypeptide N-acetylgalactosaminyltransferase-6 expression independently predicts poor overall survival in patients with lung adenocarcinoma after curative resection

**DOI:** 10.18632/oncotarget.9810

**Published:** 2016-06-03

**Authors:** Zhi Li, Sohsuke Yamada, Ying Wu, Ke-Yong Wang, Yun-Peng Liu, Hidetaka Uramoto, Kimitoshi Kohno, Yasuyuki Sasaguri

**Affiliations:** ^1^ Department of Medical Oncology, The First Hospital, China Medical University, Shenyang, China; ^2^ Department of Pathology and Cell Biology, School of Medicine, University of Occupational and Environmental Health, Kitakyushu, Fukuoka, Japan; ^3^ Bio-information Research Center, University of Occupational and Environmental Health, Kitakyushu, Fukuoka, Japan; ^4^ Second Department of Surgery, School of Medicine, University of Occupational and Environmental Health, Kitakyushu, Fukuoka, Japan; ^5^ The President Laboratory, University of Occupational and Environmental Health, Kitakyushu, Fukuoka, Japan; ^6^ Laboratory of Pathology, Fukuoka Wajiro Hospital, Fukuoka, Japan; ^7^ Division of Thoracic Surgery, Saitama Cancer Center, Saitama, Japan; ^8^ Asahi-Matsumoto Hospital, Kitakyushu, Japan; ^9^ Institute of Pathology, Medical University of Graz, Graz, Austria; ^10^ Department of Pathology, Field of Oncology, Graduate School of Medical and Dental Sciences, Kagoshima University, Kagoshima, Japan

**Keywords:** GalNAc-T6, glycosylation, lung adenocarcinoma, prognosis, overall survival

## Abstract

**Background:**

Polypeptide N-acetylgalactosaminyltransferases (GalNAc-Ts) are important glycosyltransferases in cancer, but the clinical role of its individual isoforms is unclear. We investigated the clinical significance and survival relevance of one isoform, GalNAc-T6 in lung adenocarcinoma after curative resection.

**Results:**

GalNAc-T6 was identified in 27.8% (55/198) of patients, and statistically indicated advanced TNM stage (*P* = 0.069). Multivariate analysis showed GalNAc-T6 to be an independent predictor for reduced overall survival of patients (*P* = 0.027), and the result was confirmed with bootstraping techniques, and on line “Kaplan-Meier Plotter” and “SurvExpress” database analysis, respectively. Moreover, ROC curve demonstrated that GalNAc-T6 expression significantly improved the accuracy of survival prediction.

**Methods:**

With 198 paraffin-embedded tumor samples from lung adenocarcinoma patients, GalNAc-T6 expression was immunohistochemically assessed for the association with clinicopathological parameters. The prognostic significance was evaluated by Cox proportional hazards regression analysis with 1000 bootstraping. “Kaplan-Meier Plotter”, “SurvExpress” database analysis, and receiver-operating characteristic (ROC) curve were performed to provide further validation.

**Conclusions:**

GalNAc-T6 expression correlated significantly with advanced TNM stage, and independently predicted worse OS for lung adenocarcinoma.

## INTRODUCTION

Non-small-cell lung cancer (NSCLC) remains the leading cause of cancer death worldwide [1]. Adenocarcinomas are the most common NSCLC with heterogeneous clinicopathological and molecular features [2]. More than 20% of early-stage patients would die of recurrence and metastasis [3], yet the others with low recurrence risk and long lifetime have to receive expensive, unnecessary and potentially dangerous adjuvant therapy. All these imply that current survival prediction systems based on anatomical TNM classification, histopathological features and some molecular biomarkers are deficient and need to be improved [4]. Dozens of molecules and pathways have been found to influence patients' clinical outcome. However, the clinical application of them is limited for the lack of integration of biological and clinical data and the low reproducibility of distinct studies [5]. It is of great importance to find some novel and well-validated prognostic factors.

Polypeptide N-acetylgalactosaminyltransferases (GalNAc-Ts) are the irreplaceable glycosyltransferases that initiate and catalyze the synthesis of Carbohydrate antigens (CAs) [6, 7], which include some commonly used serum tumor markers, such as CA125 and CA19-9, for the evaluation of tumor burden, disease progression in various cancers including lung adenocarcinoma [6, 8–12]. Altered expression of GalNAc-Ts is reported useful for prognostic evaluation in a large number of cancers [13–19]. We previously reported low expression of GalNAc-T3 to be an independent predictor of poor prognosis and early recurrence in lung adenocarcinoma [17].

GalNAc-T6 is highly similar to GalNAc-T3, but distinct from the other GalNAc-Ts, in gene sequence, kinetic properties and acceptor substrate specificities [20]. We have shown GalNAc-T6 positive expression to significantly correlate with good differentiation, small tumor size, absence of vascular invasion (VI), and low pTNM stage in pancreatic cancer, and lower Fuhrman's grade, absence of VI, presence of necrosis in renal cancer [18, 21]. Therefore, GalNAc-T6 is an intriguing marker of tumor behavior. However, there is still no study for the relationship of GalNAc-T6 expression with clinicopathological parameters or prognosis in lung adenocarcinoma.

Here, based on our immunohistochemical staining of 198 resected lung adenocarcinoma specimens, expression of GalNAc-T6 was revealed in one third of cases, and independently predicted shorter overall survival (OS). Moreover, the predictive validity of GalNAc-T6 for reduced OS in lung adenocarcinoma was fully verified with internal resampling and some external online database.

## RESULTS

### Patient characteristics

As shown in Table 1, the cohort included 198 patients (107 male, 91 female) with clinicopathological features representative of lung adenocarcinoma. Pathological reports were reviewed to identify patients who underwent lobectomy (178 patients), pneumonectomy (5 patients), partial resection (6 patients), or segmentectomy (9 patients) with complete mediastinal lymph node dissection for lung adenocarcinoma, respectively. The median age at surgery was 68 years. Median tumor size was 2.35 cm with a range from 0.6 to 13.5 cm. Based on American Joint Committee on Cancer criteria, the majority of patients (149 cases, 75.3%) had stage I disease, thirty-eight cases (19.2 %) had lymph node metastasis, and no one had distant metastasis at diagnosis.

**Table 1:** Characteristics of the lung adenocarcinoma study cohort

Characteristics	Number of patients (%)
Age (years)	
Median (range)	68.0 (23.0–86.0)
Gender	
Female	91 (46.0)
Male	107 (54.0)
Smoking	
Never	85 (42.9)
Ever	42 (21.2)
Current	71 (35.9)
Differentiation	
Well	87 (43.9)
Moderate	86 (43.4)
Poor	25 (12.6)
Size (cm)	
Median (range)	2.35 (0.60-13.50)
T stage	
T1	131 (66.2)
T2	53 (26.8)
T3	7 (3.5)
T4	7 (3.5)
N stage	
N0	160 (80.8)
N1	18 (9.1)
N2	20 (10.1)
TNM Stage	
I	149 (75.3)
II	21 (10.6)
III	28 (14.1)

### GalNAc-T6 expression in normal tissue and lung adenocarcinoma specimens

**Figure 1:**
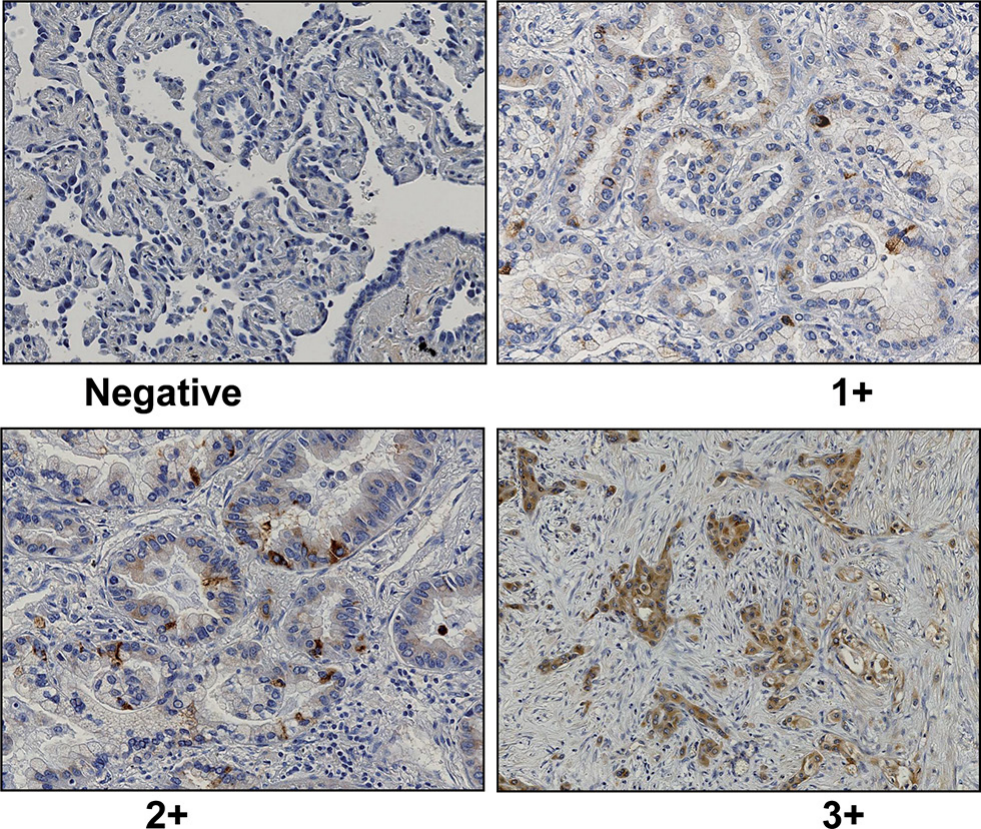
Representative data of GalNAc-T6 immunohistochemical staining (1 : 1000 dilution) in lung adenocarcinoma (× 200) Negative (A), weak (B), moderate (C), and strong (D) was shown. Immunoreactivity was observed in the cytoplasm of cancer cells.

Immunohistochemically, GalNAc-T6 expression was rare or very weak in the normal bronchioloalveolar epithelium samples (data not shown), whereas cytoplasmically immunostained in lung adenocarcinoma cells (Figure 1). As shown in Table 2, the 198 patients were divided into two groups, namely the GalNAc-T6-negative group (143 patients) and the GalNAc-T6-positive group (55 patients: 45 1+, 8 2+ and 2 3+).

**Table 2:** Association of GalNAc-T6 expression with clinicopathologic parameters

Characteristics	Negative expression of T6, *n* (%)	Positive expression of T6, *n* (%)	*P*-value
Age (years)			0.717
Median (range)	68 (23–86)	68 (40–85)	
Gender			0.386
Female	63 (44.1)	28 (50.9))	
Male	80 (55.9)	27 (49.1))	
Smoking			0.846
Never	60 (42.0)	25 (45.5))	
Ever	30 (21.0)	12 (21.8)	
Current	53 (37.1)	18 (32.7)	
Differentiation			0.028
Well	64 (44.8)	23 (41.8)	
Moderate	56 (39.2)	30 (54.5)	
Poor	23 (16.1)	2 (3.6)	
LogMax.size			0.274
Median (range)	0.83 (−0.51–2.23)	0.88 (−0.36–2.60)	
T stage			0.487
T1	94 (65.7)	37 (67.3)	
T2	37 (25.9)	16 (29.1)	
T3–T4	12 (8.4)	2 (3.6)	
N stage			0.272
N0	116 (81.1)	44 (80.0)	
N1	15 (10.5)	3 (5.5)	
N2	12 (8.4)	8 (14.5)	
TNM Stage			0.069
I	107 (74.8)	42 (76.4)	
II	19 (13.3)	2 (3.6)	
III	17 (11.9)	11 (20.0)	

### Association of GalNAc-T6 expression with clinicopathological variables

There was no significant difference between patients with distinct GalNAc-T6 expression levels regarding patients' gender, age, smoking status, size, lymph node involvement and pTNM stage. However, GalNAc-T6 expression was closely related to tumor differentiation (*P* = 0.028), and more observed in moderate differentiation tumors, and borderline significantly associated with advanced TNM stage (*P* = 0.069) (Table 2).

### Influence of GalNAc-T6 expression on survival

**Figure 2:**
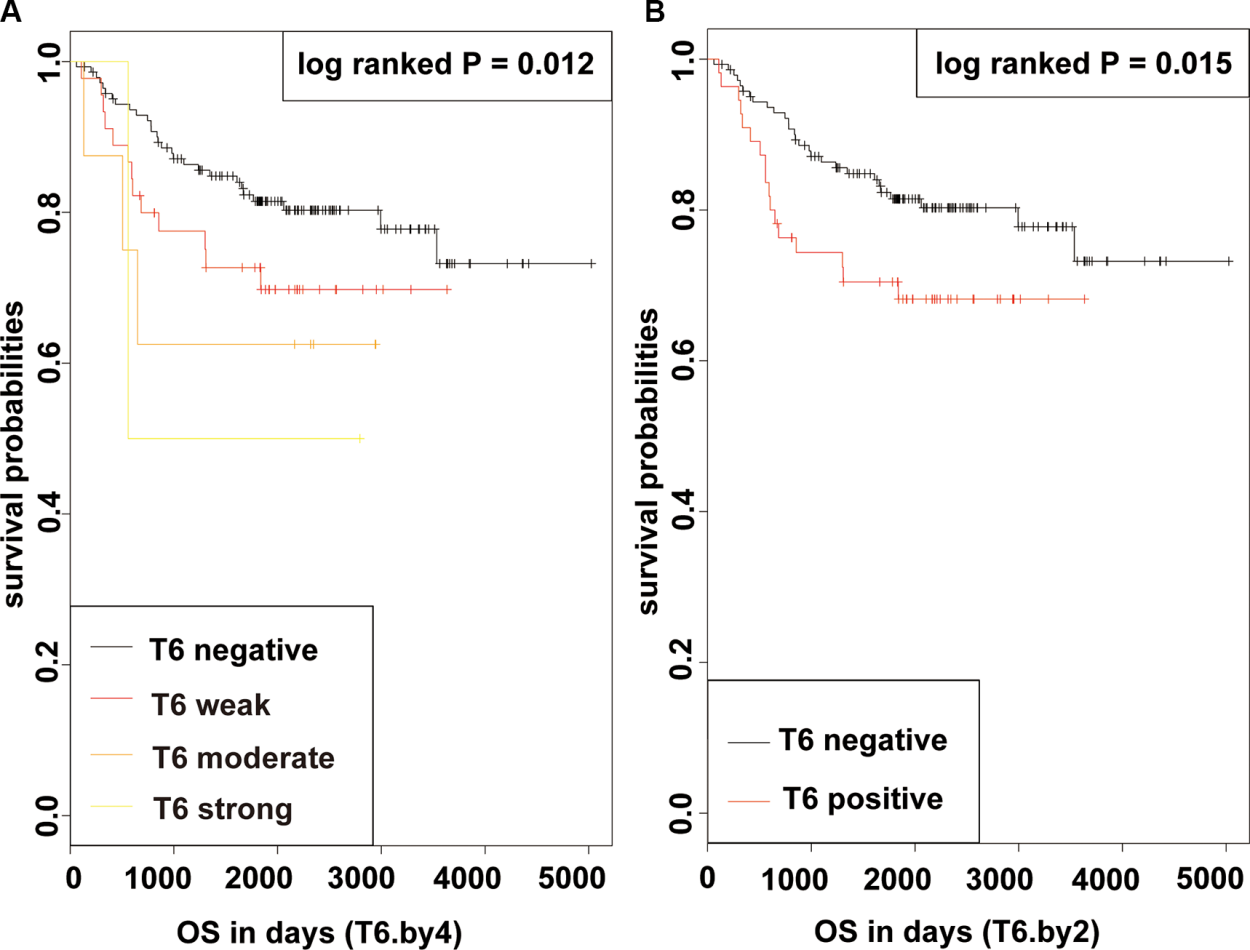
KM curves of OS in patients with lung adenocarcinoma after surgery according to GalNAc-T6 (log-rank *P* = 0.012 and 0.015 for quartered (A) and dichotomous (B) modeling of GalNAc-T6 expression, respectively)

**Figure 3:**
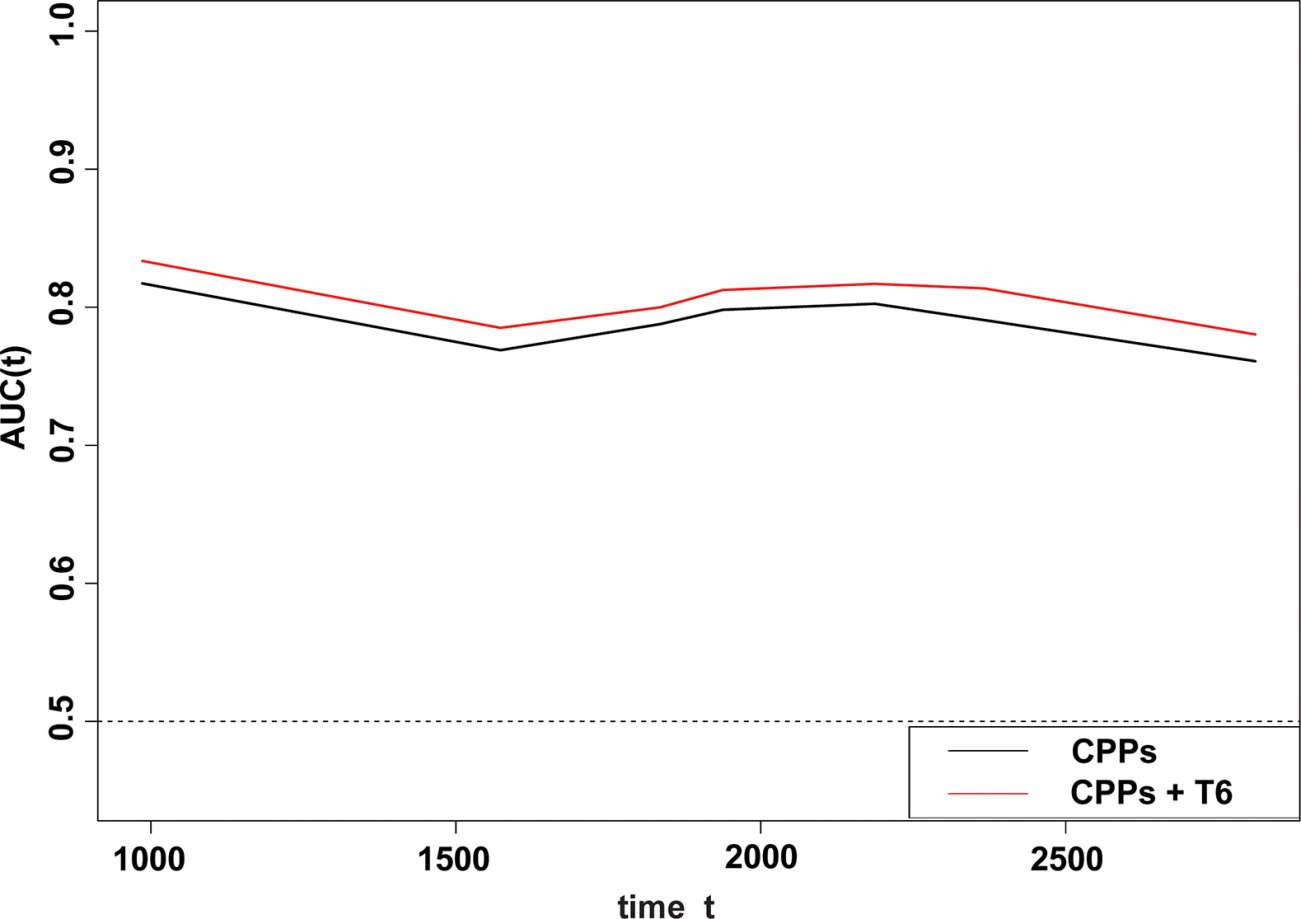
Time-dependent ROC analyses for the CPPs (gender, logMS.q, and stage), and the combination of GalNAc-T6 and CPPs The inclusion of the GalNAc-T6 expression score in the model improved the predictive ability slightly. The detailed AUC values were listed year by year in the Table S1.

In a Kaplan-Meier analysis, patients with increased GalNAc-T6 expression were usually demonstrated shorter OS irrespective of the cutoff chosen (log-rank *P* = 0.012 and 0.015 for quartered and dichotomous modeling of GalNAc-T6 expression, respectively) (Figure 2). In univariate analysis using COX proportional-hazards models, male, positive smoking history, poor differentiation, increased tumor size, advanced T stage, presence of Lymph Node (LN) metastasis, advanced pTNM stage, and enhanced GalNAc-T6 expression were revealed to indicate reduced OS (*P* = 0.007, 0.002, 0.006, < 0.001, < 0.001, < 0.001, < 0.001, and 0.027, respectively) (Table 3). The further multivariate COX PH analysis and 1000-times bootstrapping identified GalNAc-T6 to be an independent predictor of poorer OS (HR = 1.60, *P* = 0.027) (Table 3). The model diagnostics, including PH assumption, log-linearity assumption, and potential influential observation points, were further described in Supplementary Files and Figure S1 and S2. According to the time-dependent receiver-operating characteristics (ROC) curves, the inclusion of the GalNAc-T6 expression score in the model improved the predictive ability lightly (Figure 3). At the 5th year, the value of the area under the curve (AUC) was 0.798 for the model without GalNAc-T6 expression score and 0.811 for the one including the GalNAc-T6 expression score (Table S1), respectively.

**Table 3:** Univariate and multivariate analyses of OS according to clinicopathologic parameters and GalNAc-T6 expression with 1000 bootstraping

Characteristics	No		Uni-variant analysis			Multi-variant analysis			
	Patients	Events	HR	95% CI	*P*-value	HR	95% CI	*P*-value	Bootstrapping 95% CI
Age (years)	-	-	1.017	0.987–1.048	0.264				
Gender			2.427	1.273–4.628	0.007	2.76	1.418–5.369	0.003	1.492–5.697
Female	91	13							
Male	107	32							
Smoking				1.723	1.222–2.430	0.002			
Never	85	11							
Ever	42	10							
Current	71	24							
Differentiation				1.791	1.187–2.702	0.006			
Well	87	11							
Moderate	86	27							
Poor	25	7							
LogMax.size			2.290	1.655–3.169	0.000	2.03	1.450–2.849	0.000	1.487–2.959
0–25%	50	3							
26–50%	49	6							
51–75%	56	15							
76–100%	43	21							
T stage				2.709	1.840–3.988	0.000			
T1	131	15							
T2	53	23							
T3–T4	14	7							
N stage				1.923	1.349–2.741	0.000			
N0	160	30							
N1	18	4							
N2	20	11							
TNM Stage			2.098	1.519–2.898	0.000	1.92	1.344–2.743	0.000	1.319–2.886
I	149	22							
II	21	10							
III	28	13							
T6			1.569	1.051–2.342	0.027	1.60	1.054–2.417	0.027	1.071–2.494
–	143	28							
+	45	13							
++	8	3							
+++	2	1							

### External validation of findings

Patients with high level of GalNAc-T6 showed significantly shorter OS than those with low expression (*P* = 0.0074), according to the Kaplan-Meier Plotter database (Figure S3). In addition, we investigated the prognostic value of GalNAc-T6 using another web- based system, SurvExpress. The pooled HR (95% CI) of GalNAc-T6 was 1.15 (0.942–1.391) and 1.31 (0.951– 1.804) in the fixed and random effect model analysis, respectively (Table S2, Figure S4).

### Subgroup analysis

In the patients with postoperative therapy, overexpression of GalNAc-T6 significantly indicated reduced survival (*P* = 0.017) (Figure S5 left). In the subgroup without postoperative therapy, the curve represented positive expression of GalNAc-T6 was much lower, but with a statistically insignificant *P* value (*P* = 0.203) (Figure S5 right)

## DISCUSSION

For the past few years, the roles of GalNAc-Ts in cancers have been explored by variety of molecular and clinical studies. GalNAc-Ts were shown to increase cellular proliferation, suppress apoptosis, and enhance migration invasion in several cancer types [22–27]. Clinically, GalNAc-T2 and -T9 predict favorable prognosis in neuroblastoma [14, 26]. In renal cancer, low GalNAc-T4, or high GalNAc-T3, -T6, and -T10 indicates poor survival and early cancer recurrence [21, 28, 29]. In gastric cancer, low GalNAc-T5 is associated with poor prognosis [30]. Here with IHC staining, GalNAc-T6 was revealed to express in a third of our lung adenocarcinoma specimens, statistically associated with tumor differentiation and borderline significantly with advanced pTNM stage. Further Kaplan-Meier and univariate/multivariate COX analysis indicated GalNAc-T6 to be the independent predictor for reduced survival. Moreover, based on ROC curve, GalNAc-T6 strengthens the predictive efficacy of traditional clinicopathological features in lung adenocarcinoma.

Our finding is credible and generally applicable on account of the following evidences. First, GalNAc-T6, regardless of the IHC cutoff value selected, consistently indicated reduced OS in lung adenocarcinoma. Second, the independent prediction of GalNAc-T6 for OS was determined by COX regression model that was proved robust by the proportional hazards (PH) assumption and influential observation diagnostics; and further confirmed by 1000 internal bootstrap replications and 2 distinct external validations using online high-throughput datasets. Third, such correlation of GalNAc-T6 with poor prognosis was previously reported in breast cancer, where higher GalNAc-T6 mRNA in bone marrow signified recurrence [19]; and in gastric cancer, where strong GalNAc-T6 expression correlated with VI [31].

The indicative role of GalNAc-T6 for poor prognosis might be explained molecularly by some past and recent findings. Epithelial-to-mesenchymal transition (EMT) is the critical process for tumor metastasis [32], and could be suppressed by inhibiting mucin-1 (MUC1) glycosylation [33]. GalNAc-T6 was reported to induce EMT-like changes by mediating MUC1 glycosylation in breast cancer [34, 35], and promote EMT in prostate cancer cells treated by transforming growth factor-beta [36]. Thus, GalNAc-T6 might lead to a poor prognosis in lung adenocarcinoma through promoting EMT-related metastasis, which deserves further experiments.

Contradictorily, GalNAc-T6 predicted poor prognosis in the current lung adenocarcinoma and our previous renal cancer study [28], whereas indicated good survival in pancreatic cancer reported by us [18], and significantly more often observed in early stage and good differentiated breast tumor [37, 38]. The similar conflict was also revealed by the studies of other GalNAc-Ts. GalNAc-T3 is revealed the independent predictor for reduced disease-free survival in early stage oral squamous cell carcinoma, and poor prognosis in renal cell carcinomas, respectively [21, 39], but indicated reduced invasive depth and good prognosis in lung adenocarcinoma and colon cancer [17, 31]. GalNAc-T2 enhanced migration and invasion of oral squamous cell carcinoma [25], but predicted favorable prognosis in neuroblastoma [26]. Up to now, there is no reasonable explanation and direct experiment for the contradictory influence of the individual GalNAc-T isoform in different cancers. Our speculations are as follows: 1) there are the site-specific protein O-glycosylation, the different repertoire and substrate proteins of GalNAc-Ts isoforms in different cancers [8, 40, 41]. 2) GalNAc-Ts including -T6, might appear as the early whereas not persist event with tumor progression in some cancers, but as a late event in other cancer types. Further investigations are warranted in future.

It was noticed that the 5-year survival rate of our cohort was 73.0%, which seemed higher comparing with other lung adenocarcinoma studies, but could be explained by the percentage of our patients with early pTNM stages. Of the 198 cases, 149 were in stage I (75.3%), 21 in stage II (10.6%), and only 28 in stage III (14.1%). In the stage I group, 117 patients were in stage Ia, and 32 in stage Ib, and their 5-year survival rates were 89.8% and 67.2%, respectively. Similarly, the 5-year survival rates for stage I patients in some large Japanese lung cancer cohorts were 66.3 – 89.3% [42, 43]. Therefore, our cohort could be considered representative for Japanese lung adenocarcinoma population. Given that our results were also validated with the online database including studies mostly from Europe and America, the prognostic value of GalNAc-T6 should be contributable for not only the population of Japan.

In summary, GalNAc-T6 presence in lung adenocarcinoma is closely related with tumor differentiation, borderline significantly indicated advanced stage, and independently predicted reduced OS of patients. Since most conventional serum markers are CAs produced from protein glycosylation catalyzed by GalNAc-Ts [44, 45], GalNAc-T6 is a candidate biomarker could be applied clinically in combination with these conventional tumor markers. Given that distinct GalNAc-Ts family members locate in different cellular compartments, display tissue-specific expression, and have different but partly overlapping functions [46], further exploration are thus warranted for the molecular function and the competitive or complementary correlation of distinct GalNAc-T isozymes in lung adenocarcinoma, and the prognostic value of individual GalNAc-Ts or those combined with the conventional serum biomarkers.

## MATERIALS AND METHODS

### Patients and tumor specimens

In accordance with the reporting recommendations for tumor marker prognostic studies (REMARK) criteria [47] and the guidelines of the Japan Society of Pathology, this retrospective study was performed with the approve of the institutional review board of the University of Occupational and Environmental Health (UOEH). The use of specimens from human subjects was authorized by written consent from next of kin, and the patient records/information was anonymized and de-identified prior to analysis.

Primary lung adenocarcinoma (pathologic stages I-III) samples received a complete resection from 1997 to 2005 were reviewed, and totally 258 patients were registered. Of them, 60 patients were excluded because of 1) preoperative radiotherapy/chemotherapy (9 cases); 2) another malignancy except for basal cell skin carcinoma and stage I cervical cancer (14 cases); 3) unclear margins by microscopic examination (25 cases); 4) perioperative death happened during the patient's initial hospitalization or within 30 days of surgery (3 cases); 5) inadequate paraffin-embedded fixed tissue blocks (5 cases); or 6) incomplete clinical/pathologic data (2 cases). Thus, 198 patients with complete medical records and adequate paraffin-embedded tissue blocks were eligible.

Totally, 27 (13.6%) patients received post-operative adjuvant chemotherapy as follows: 18, carboplatin plus paclitaxel; 7, carboplatin plus gemcitabine; and 2, tegafur-uracil. No patient was treated with EGFR-TKI. Patients underwent chest x-rays and blood chemistry every month during the first 3 years and every 3 months thereafter. Computed tomography, bone scintigram, and brain magnetic resonance imaging were performed every 6 months. This report includes follow-up data as of December 14, 2010, and the median follow-up time, as calculated by the reverse Kaplan-Meier method [48], was 2190 days. OS was set on the period from the date of surgery to death or the most recent clinic visit.

### IHC assay and criteria

Formalin-fixed paraffin-embedded (FFPE) tumor specimens were obtained from the archives of the Department of Pathology at UOEH hospital. Three pathologists examined all resected specimens to confirm the histopathological features. The tumors were staged according to the tumor-node-metastasis system of the American Joint Committee on Cancer, and histologically subtyped and graded according to World Health Organization guidelines and General Rule for Clinical and Pathological Record of Lung Cancer (7th Edition). Normal human tissue was obtained from non-tumor portion of surgically resected specimens.

GalNAc-T6 was detected by immunohistochemistry (IHC) as described previously [18]. Less than 10% of positivity was considered as negative. Staining equal to or more than 10% were defined as positive staining and were graded into three categories: 1+, positivity of 10–30%; 2+, 30–80%; 3+, more than 80%. All histological and IHC slides were evaluated by two independent observers (certified surgical pathologists in our department: Li Zhi and Sohsuke Yamada) who were blinded to the clinicopathological data. Based on the interclass correlation coefficient, the agreement between observers was excellent (> 0.9) for all antibodies. For the few instances of disagreements, the third board-certified pathologist in our department (Yasuyuki Sasaguri) determined the consensus scores.

### Statistical analysis

Expression level of GalNAc-T6 was analyzed as a dichotomous variable (negative VS positive). Gender, smoking, differentiation, T-stage, and N-stage were considered as categorical variables. Age and max-size of tumor were measured as continuous variables. In COX PH survival analysis, the log-transformed variable tumor size (logMax.size) was quartered and thus transformed to categorical variable. The associations of GalNAc-T6 with categorical variables were tested with Chi-square test or the Fisher exact test, as appropriate. Welch's two-sample *t*-test was used to compute *P* values for continuous variables.

Kaplan–Meier curves and log-rank tests were used for survival analysis. Univariate associations between OS and GalNAc-T6 as well as other clinicopathological parameters (CPPs) were examined using Cox PH regression model, with hazard ratios (HRs) and 95% confidence interval (CI) being calculated alongside.

Multivariable Cox regression models were adjusted by all the other CPPs (age, gender, smoking, differentiation, logMax.size, T-stage, and N-stage). GalNAc-T6 was modeled as dichotomous, or quartered, according to the relative fit of multivariate models adjusted for the standard prognostic factors, and assessed using likelihood ratios and Akaike Information Criterion (AIC). A backward selection was then applied to construct the final multivariate model based on the AIC value. The Schoenfeld residual test (cox.zph function in R) was used to test the PH model assumption whether the log relative hazard is constant over time, with a *p* value < 0.05 as a violation [49]. The assumptions of the model, including log-linearity assumption and potential influential observation, were verified by graphical methods [49]. Internal validation of the Cox model was performed using bootstrapping (1000 replications). The covariable coefficients, including the 95% CI, were thus estimated.

To evaluate the prediction accuracy of GalNAc-T6 in the Cox model, time-dependent receiver-operating characteristic (ROC) curves for censored data and resulting area under the curve (AUC) were constructed according to Heagerty et al. [50]. The risk scores to generate time-dependent sensitivity and specificity for the corresponding ROC curve at each observed event time were calculated. The AUC (t) curve was plotted to assess the prediction accuracy of the model.

To externally validate the prognostic value of GalNAc-T6, we used the Kaplan-Meier Plotter database analysis [51]. OS was assessed in lung adenocarcinoma patients stratified by median GalNAc-T6 expression. All other parameters were left at default settings, except for “treatment group”, which was set as “only surgical margins negative”, to simulate the current cohort maximally. In addition, we used “SurvExpress”, an online biomarker validation tool to perform survival analysis [52]. Moreover, five of 22 published lung cancer studies were for patients with pure adenocarcinoma. With the default settings, we extracted study name, sample size, HR and 95% CI of the five studies for further meta-analysis. The detailed meta-analysis steps were described in the Supplementary Files.

To evaluate the prognostic value of GalNAc-T6 according to the adjuvant chemotherapy status, we perform a subgroup analysis with Kaplan–Meier curves and log-rank tests.

All statistical tests were two-tailed with a *P* < 0.05 considered significant, and a *P* < 0.10 considered borderline significant. R software (version 3.1.1) was used for the above statistical analysis.

## SUPPLEMENTARY MATERIALS


