# Supplementary material for: Polypeptide N-acetylgalactosaminyltransferase-6 expression independently predicts poor overall survival in patients with lung adenocarcinoma after curative resection
Source: Oncotarget. 2016 Jun 3;7(34):54463–73. doi: 10.18632/oncotarget.9810 (PMC5342355; doi:10.18632/oncotarget.9810)
Supplement: Supplementary file 1 [file oncotarget-07-54463-s001.pdf]

Polypeptide N-acetylgalactosaminyltransferase-6 expression independently predicts poor overall survival in patients with lung adenocarcinoma after curative resection

Supplementary Materials

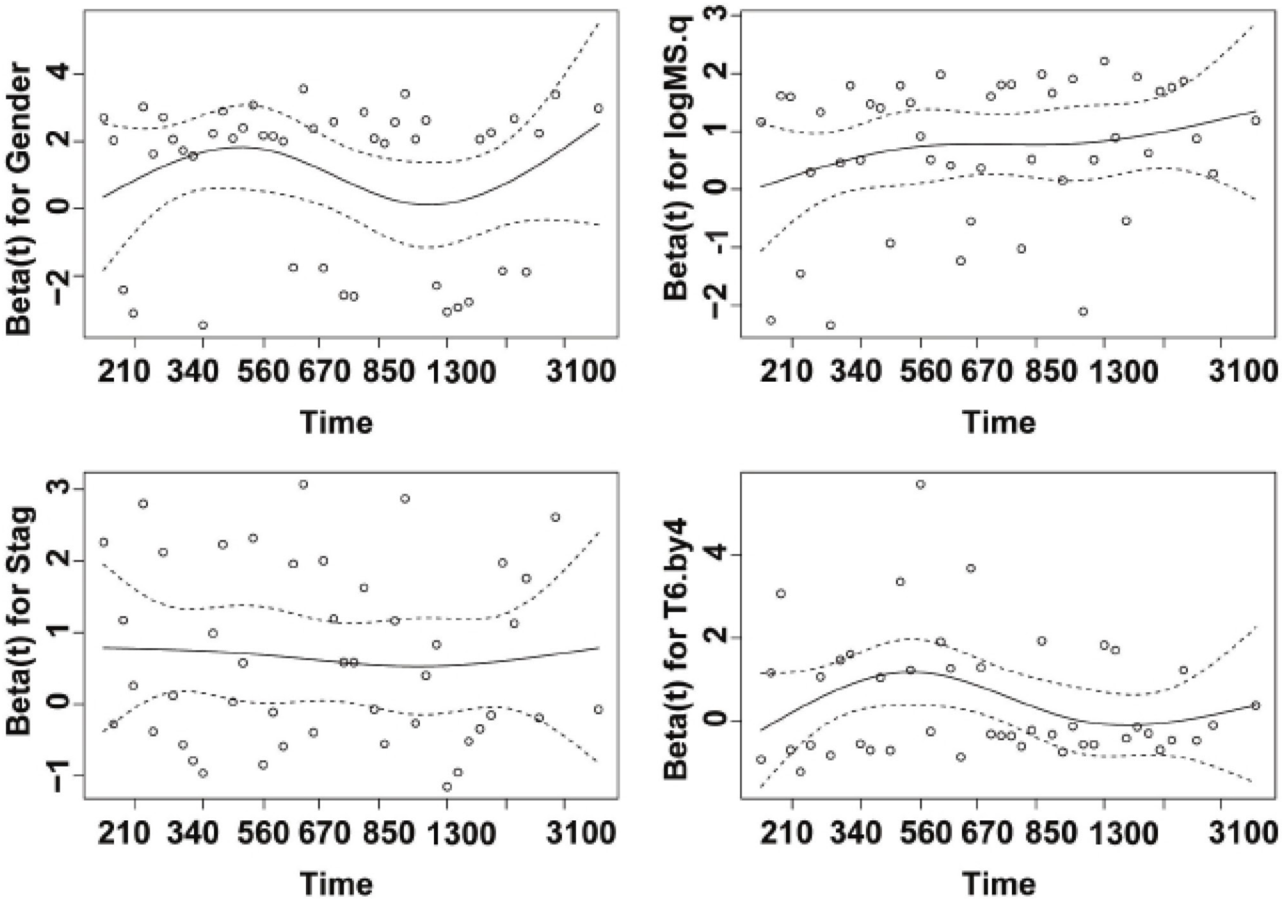

Supplementary Figure S1: Plots of scaled schoenfeld residuals against transformed time for each covariate in the final Cox model.

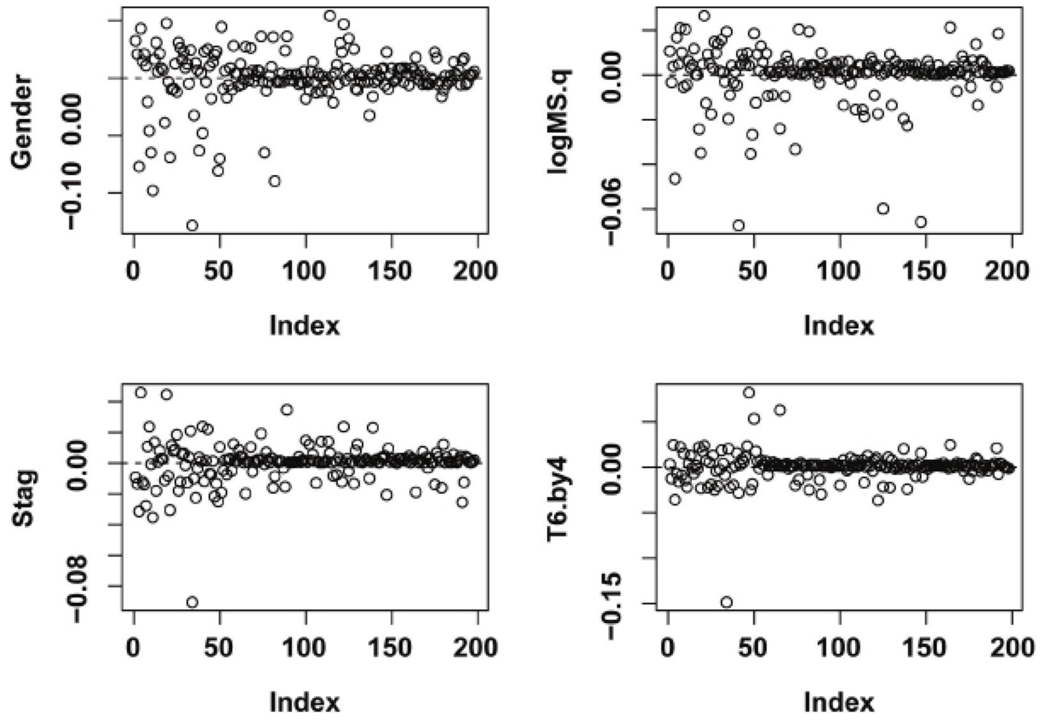

Supplementary Figure S2: Index plots of dfbeta for the Cox regression of time to rearrest on each variable in the final Cox model.

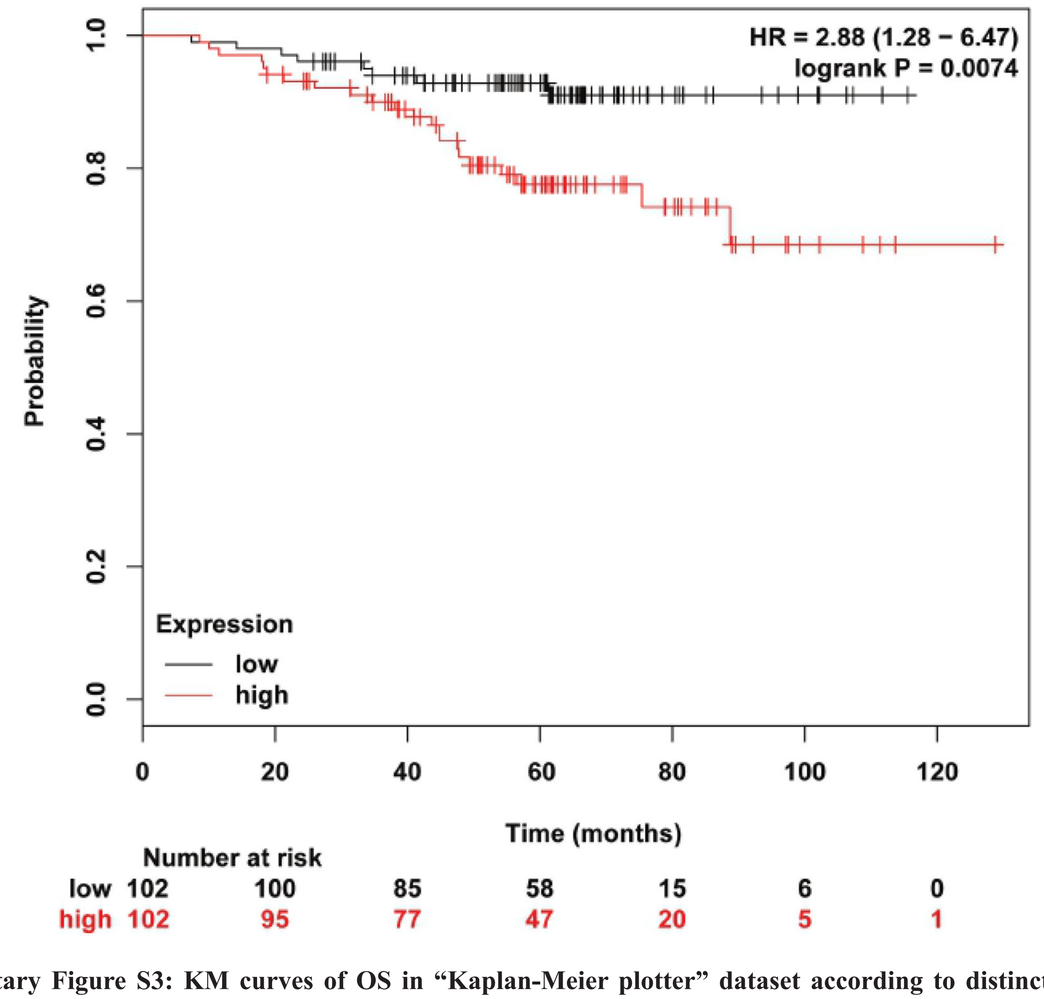

Supplementary Figure S3: KM curves of OS in "Kaplan-Meier plotter" dataset according to distinct GalNAc-T6 expression level.

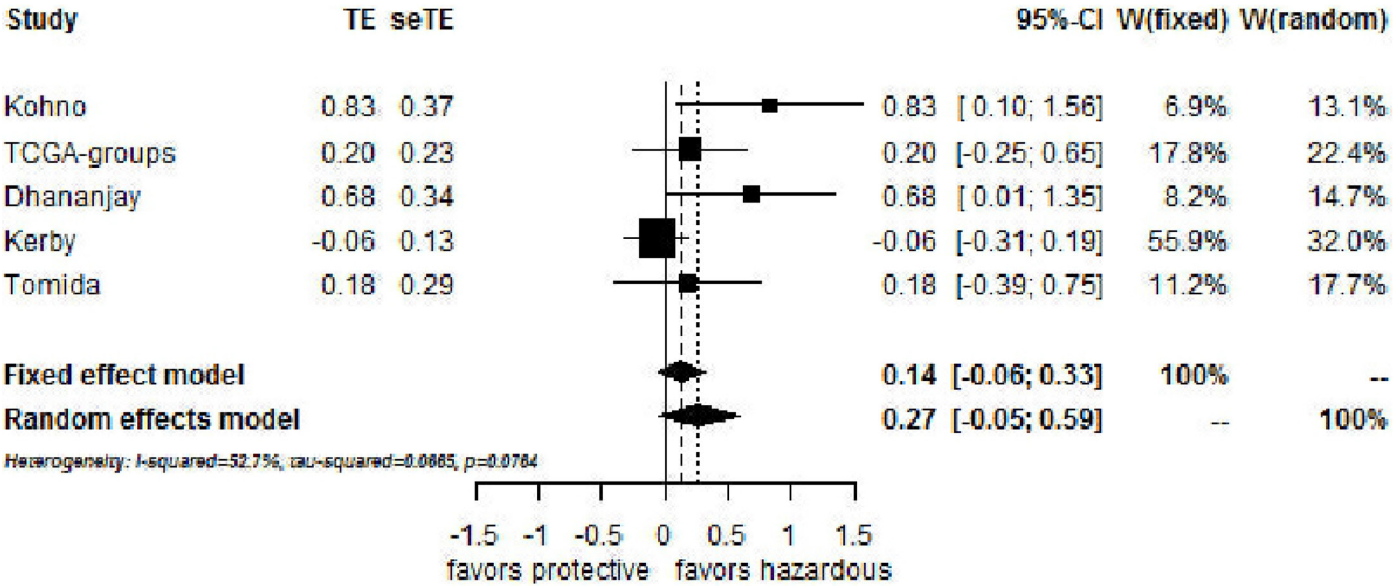

Supplementary Figure S4: Meta-analysis of the association between GalNAc-T6 overexpression and OS of lung adenocarcinoma studies in the "SurvExpress" datasets.

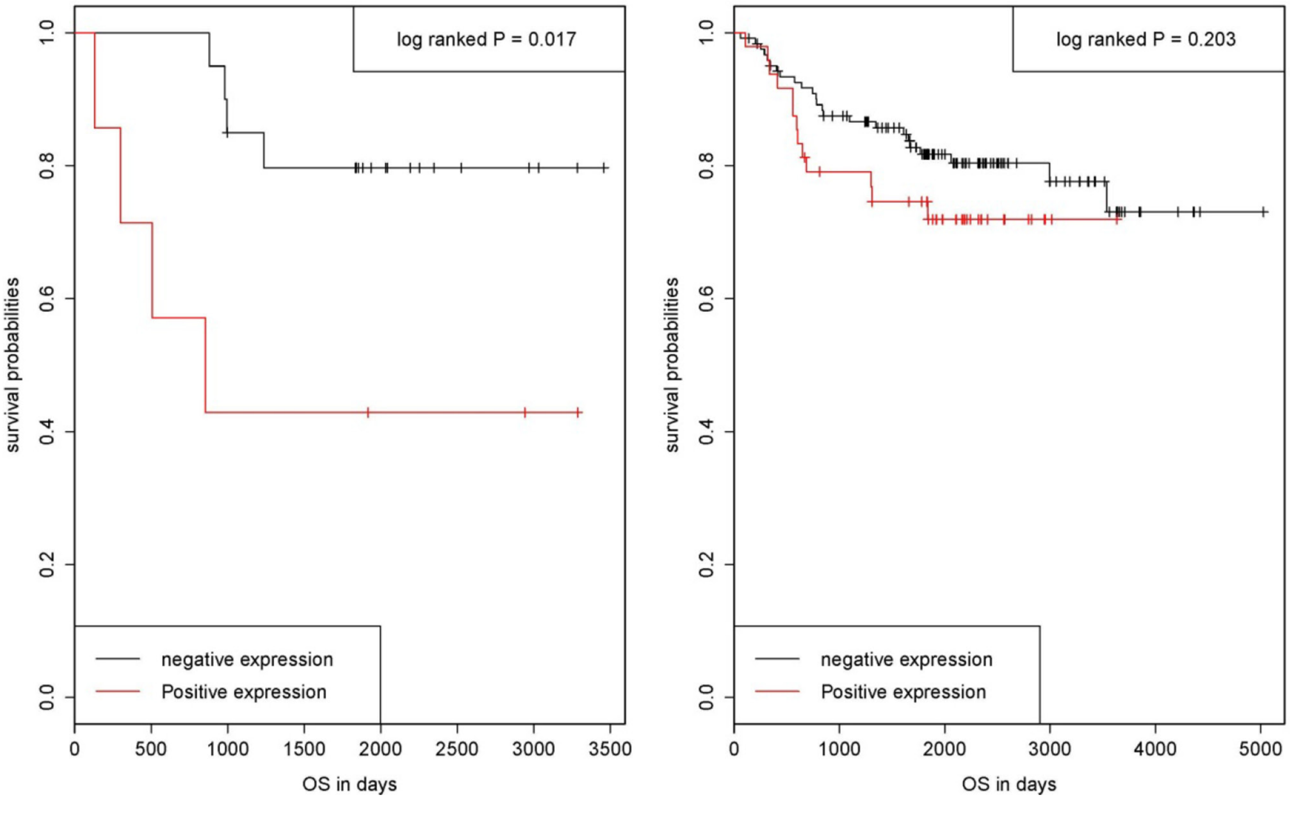

Supplementary Figure S5: KM curves of OS in different postoperative therapy status according to distinct GalNAc-T6 expression level. Left is for patients with postoperative therapy, and the log-rank $P = 0.017$; right is for patients without postoperative therapy, and the log-rank $P = 0.203$.

Supplementary Table S1: Time-dependent ROC analysis of the CPP*s (gender, logMS.q, and stage), and the combination of GalNAc-T6 and CPPs annually

AUC	1st Year	2nd Year	3rd Year	4th Year	5th Year
CPPs + GalNAc-T6	0.712	0.815	0.842	0.790	0.811
CPPs	0.705	0.783	0.827	0.772	0.798
* represents "clinicopathological parameters"					

Supplementary Table S2: Baseline characteristics of the lung adenocarcinoma studies in the "SurvExpress" dataset

Studies	Author	Size	HR	L 95CI	U 95CI	*P* value
GSE31210	Kohno	226	2.3	1.12	4.69	0.02245
TCGA	TCGA-groups	255	1.22	0.78	1.91	0.3866
Chitale Lung	Dhananjay	185	1.97	1.02	3.82	0.04397
DCCNL	Kerby	462	0.94	0.73	1.21	0.624
GSE13213	Tomida	117	1.2	0.68	2.1	0.5308

COX PH Model diagnostics and meta analysis using data in SurvExpress database

COX PH model diagnostics

The cox.zph global *p*-value for the full model was 0.299, however, the PH assumption for covariable "logMax. size" (log-transformed tumor size) was violated ($P = 0.031$). Thus it was transformed from continuous variable to categorical by using quartile as cutoff. The final COX PH model was rebuilt and PH assumption wasn't violated any more (Figure S1). In the final model, GalNac-T6 remained to be independent prognostic factor. As in the final COX PH model, no variable was continuous, the log-linearity assumption wasn't checked. According to influential observation evaluation, the index plots appear in Figure S2. Comparing the magnitudes of the largest dfbeta values to the regression coefficients suggests that none of the observations is terribly influential individually.

Meta Analysis using data in SurvExpress database

As mentioned in the main text, five articles were included in the meta analysis, and the study names, sample size, HR and 95% CI were extracted and listed in Table S2. Meta analysis was performed with R software. The pooled HR with a 95%CI was obtained by calculating a weighted average of the individual log(HR) estimates. A pooled HR > 1 implied a worse survival for the group with GalNAc-T6 high expression. Data were considered statistically significant if the 95% CI for the combined HR did not overlap 1.

The final results were shown in Table S2 and Figure S4. The pooled HR of GalNAc-T6 was 1.15 (0.942–1.391) and 1.31 (0.951–1.804) in the fixed and random effect model analysis, which was transformed from lnHR and its 95% CI.
